# Multisite implementation of a workflow-integrated machine learning system to optimize COVID-19 hospital admission decisions

**DOI:** 10.1038/s41746-022-00646-1

**Published:** 2022-07-16

**Authors:** Jeremiah S. Hinson, Eili Klein, Aria Smith, Matthew Toerper, Trushar Dungarani, David Hager, Peter Hill, Gabor Kelen, Joshua D. Niforatos, R. Scott Stephens, Alexandra T. Strauss, Scott Levin

**Affiliations:** 1 Department of Emergency Medicine, Johns Hopkins University School of Medicine, Baltimore, MD USA; 2 Malone Center for Engineering in Healthcare, Johns Hopkins University Whiting School of Engineering, Baltimore, MD USA; 3 Center for Disease Dynamics, Economics & Policy, Washington, DC USA; 4 Department of Medicine, Howard County General Hospital, Columbia, MD USA; 5 Department of Medicine, Johns Hopkins University School of Medicine, Baltimore, MD USA

**Keywords:** Health services, Prognosis, Translational research

## Abstract

Demand has outstripped healthcare supply during the coronavirus disease 2019 (COVID-19) pandemic. Emergency departments (EDs) are tasked with distinguishing patients who require hospital resources from those who may be safely discharged to the community. The novelty and high variability of COVID-19 have made these determinations challenging. In this study, we developed, implemented and evaluated an electronic health record (EHR) embedded clinical decision support (CDS) system that leverages machine learning (ML) to estimate short-term risk for clinical deterioration in patients with or under investigation for COVID-19. The system translates model-generated risk for critical care needs within 24 h and inpatient care needs within 72 h into rapidly interpretable COVID-19 Deterioration Risk Levels made viewable within ED clinician workflow. ML models were derived in a retrospective cohort of 21,452 ED patients who visited one of five ED study sites and were prospectively validated in 15,670 ED visits that occurred before (*n* = 4322) or after (*n* = 11,348) CDS implementation; model performance and numerous patient-oriented outcomes including in-hospital mortality were measured across study periods. Incidence of critical care needs within 24 h and inpatient care needs within 72 h were 10.7% and 22.5%, respectively and were similar across study periods. ML model performance was excellent under all conditions, with AUC ranging from 0.85 to 0.91 for prediction of critical care needs and 0.80–0.90 for inpatient care needs. Total mortality was unchanged across study periods but was reduced among high-risk patients after CDS implementation.

## Introduction

As of December 2021, there have been more than 270 million confirmed cases of severe acute respiratory syndrome coronavirus 2 (SARS-CoV-2) infection worldwide and 5.3 million deaths attributed to coronavirus disease 2019 (COVID-19)^1^. Resources required to care for this population and overwhelming demand have strained emergency and inpatient care systems across the globe^2,3^. Moreover, new highly transmissible SARS-CoV-2 variants and vaccine hesitancy have caused recurrent surges of infection and severe COVID-19 that outstrip healthcare resources (e.g., staff, physical space, ventilators)^4^. To optimize capacity, patients with and under investigation for COVID-19 must be matched to appropriate levels of care.

Emergency departments (EDs) are the primary point of access to hospital-based care and are tasked with distinguishing patients who require hospitalization from those who do not^5–7^. These determinations are often based on limited data and prior clinical experience is used to anticipate clinical trajectory. The novelty of COVID-19 and highly variable clinical courses introduce high uncertainty for this population^8^. High content analytics, applied to data collected from the thousands of patients cared for in the ED with COVID-19 to date, can be used to reduce this uncertainty and optimize resource allocation.

We describe the development and health system-wide implementation of a clinical decision support (CDS) system that uses machine learning (ML) prediction models to estimate short-term risk of clinical deterioration in patients with diagnosed or suspected COVID-19 and to optimize ED disposition decisions. This system analyzes electronic health record (EHR) data in real time. It delivers CDS in the form of COVID-19 Deterioration Risk Levels and disposition recommendations integrated within existing EHR workflow and delivered at the point of disposition decision-making.

## Results

### Cohort characteristics

**Fig. 1:**
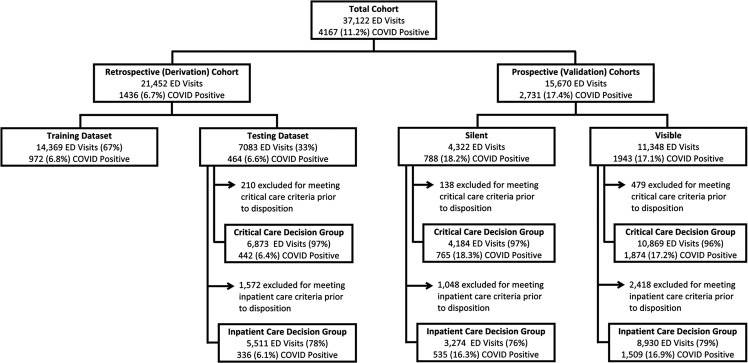
Study inclusion flowchart.

A retrospective cohort comprised of 21,452 adult ED encounters by 18,810 unique patients was used for model derivation, separated into training (67%) and testing (33%) datasets (Fig. 1). Overall incidence of critical care needs at 24 h in this cohort was 10.6% (*n* = 2265), while the overall incidence of inpatient care needs at 72 h was 22.2% (*n* = 4760); incidence was similar between derivation and validation datasets (Table 1). After excluding patients who met full or partial outcome criteria prior to ED disposition decision from the testing dataset, 6873 (97.0%) and 5511 (77.8%) encounters remained for evaluation of model performance in predicting need for critical and inpatient care, respectively (Fig. 1).

**Table 1:** Study cohort characteristics.

	Total	Retrospective derivation	Retrospective validation	Prospective silent	Prospective visible
Total visits	37,122	14,369	7083	4322	11,348
Critical Care Decision Group	35,842 (96.6)	13,916 (96.8)	6873 (97.0)	4184 (96.8)	10,869 (95.8)
Inpatient Care Decision Group	28,987 (78.1)	11,272 (78.4)	5511 (77.8)	3274 (75.8)	8930 (78.7)
Age, *N* (%)					
18–44 years	15,123 (40.7)	6046 (42.1)	2933 (41.4)	1456 (33.7)	4688 (41.3)
45–64 years	11,764 (31.7)	4416 (30.7)	2249 (31.8)	1348 (31.2)	3751 (33.1)
65–74 years	4684 (12.6)	1786 (12.4)	887 (12.5)	598 (13.8)	1413 (12.5)
>74 years	5551 (15.0)	2121 (14.8)	1014 (14.3)	920 (21.3)	1496 (13.2)
Gender, *N* (%)					
Female	19,805 (53.4)	7522 (52.3)	3766 (53.2)	2358 (54.6)	6159 (54.3)
Race/ethnicity, *N* (%)					
Black non-Latino	14,377 (38.7)	5399 (37.6)	2686 (37.9)	1331 (30.8)	4961 (43.7)
White non-Latino	14,479 (39.0)	5556 (38.7)	2688 (38.0)	1887 (43.7)	4348 (38.3)
Latino	4843 (13.0)	2192 (15.3)	1122 (15.8)	564 (13.0)	965 (8.5)
Other	3423 (9.2)	1222 (8.5)	587 (8.3)	540 (12.5)	1074 (9.5)
COVID-19 status, *N* (%)					
COVID-19 positive	4167 (11.2)	972 (6.8)	464 (6.6)	788 (18.2)	1943 (17.1)
Positive at disposition	1884 (5.1)	724 (5.0)	401 (5.7)	227 (5.3)	532 (4.7)
Chief complaint, *N* (%)					
Shortness of breath	8257 (22.2)	3190 (22.2)	1554 (21.9)	1037 (24.0)	2476 (21.8)
COVID-19 concerns	4297 (11.6)	1732 (12.1)	863 (12.2)	805 (18.6)	897 (7.9)
Chest pain	3931 (10.6)	1503 (10.5)	763 (10.8)	414 (9.6)	1251 (11.0)
Fever	2951 (7.9)	1372 (9.5)	663 (9.4)	332 (7.7)	584 (5.1)
Abdominal pain	3415 (9.2)	1211 (8.4)	605 (8.5)	406 (9.4)	1193 (10.5)
Comorbidities, *N* (%)					
Atrial fibrillation	1770 (4.8)	693 (4.8)	354 (5.0)	199 (4.6)	524 (4.6)
Coronary artery disease	2315 (6.2)	905 (6.3)	454 (6.4)	239 (5.5)	717 (6.3)
Cancer	3336 (9.0)	1225 (8.5)	645 (9.1)	322 (7.5)	1144 (10.1)
Cerebrovascular disease	1538 (4.1)	591 (4.1)	308 (4.3)	163 (3.8)	476 (4.2)
Diabetes	4009 (10.8)	1566 (10.9)	760 (10.7)	401 (9.3)	1282 (11.3)
Heart failure	2041 (5.5)	813 (5.7)	407 (5.7)	166 (3.8)	655 (5.8)
Hypertension	6887 (18.6)	2584 (18.0)	1309 (18.5)	731 (16.9)	2263 (19.9)
Immunosuppression	2650 (7.1)	1012 (7.0)	526 (7.4)	225 (5.2)	887 (7.8)
Kidney disease	3174 (8.6)	1194 (8.3)	568 (8.0)	291 (6.7)	1121 (9.9)
Liver disease	3339 (9.0)	1347 (9.4)	641 (9.0)	260 (6.0)	1091 (9.6)
Pregnancy	506 (1.4)	198 (1.4)	102 (1.4)	35 (0.8)	171 (1.5)
Prior respiratory failure	662 (1.8)	253 (1.8)	107 (1.5)	78 (1.8)	224 (2.0)
Smoker	1593 (4.3)	643 (4.5)	313 (4.4)	88 (2.0)	549 (4.8)
Vital signs, Mean (95% CI)					
Temperature, ^o^F	98.5 (96.8–101.9)	98.6 (96.8–102.0)	98.6 (96.8–102.0)	98.6 (96.8–102.0)	98.4 (96.6–101.5)
Heart rate, bpm	86.6 (56.0–125.0)	86.6 (57.0–125.0)	86.4 (56.0–126.0)	86.2 (56.0–122.0)	86.8 (56.0–126.0)
Respiratory rate, bpm	18.5 (14.0–30.0)	18.4 (14.0–30.0)	18.5 (14.0–30.0)	19.0 (14.0–32.0)	18.4 (14.0–30.0)
Oxygen saturation, %	97.4 (92.0–100.0)	97.5 (92.0–100.0)	97.5 (91.0–100.0)	97.1 (91.0–100.0)	97.4 (92.0–100.0)
Systolic blood pressure, mmHg	131.0 (95.0–184.0)	131.3 (95.0–185.0)	131.1 (95.0–183.0)	130.7 (94.4–181.6)	130.8 (94.0–184.0)
Labs, *N* tested, Mean (95% CI)					
Absolute lymphocyte Count, K/cu mm	2155, 1.9 (0.1–7.3)	669, 1.9 (0.1–7.0)	361, 2.3 (0.2–7.3)	315, 1.8 (0.1–8.4)	810, 1.9 (0.1–6.4)
Alanine aminotransferase, U/L	24,123, 36.2 (7.0–136.0)	8776, 37.7 (7.0–139.0)	4312, 35.5 (7.0–128.0)	3159, 35.0 (7.0–130.0)	7876, 35.3 (7.0–142.0)
Aspartate Aminotransferase, U/L	21,518, 40.8 (11.0–156.1)	7858, 41.9 (11.0–160.6)	3832, 38.2 (11.0–155.2)	2854, 42.3 (13.0–142.7)	6974, 40.4 (11.0–159.0)
Bilirubin, mg/dl	24,616, 0.6 (0.0–1.9)	8959, 0.6 (0.0–2.0)	4414, 0.6 (0.0–2.0)	3193, 0.6 (0.0–1.8)	8050, 0.6 (0.0–1.8)
Blood urea nitrogen, mg/dL	25,212, 18.5 (5.0–64.7)	9165, 18.3 (5.0–63.0)	4542, 18.8 (5.0–66.5)	3298, 19.5 (6.0–68.0)	8207, 18.1 (6.0–63.0)
Creatinine, mg/dL	25,212, 1.3 (0.5–5.2)	9165, 1.2 (0.5–4.9)	4542, 1.3 (0.5–5.8)	3298, 1.2 (0.5–4.9)	8207, 1.3 (0.5–5.4)
C-Reactive protein, mg/dL	1792, 19.5 (0.0–160.0)	698, 19.3 (0.0–159.9)	326, 21.9 (0.0–178.9)	306, 29.2 (0.0–168.7)	462, 11.9 (0.0–105.8)
D-dimer, mg/L	5625, 1.3 (0.0–8.4)	1851, 1.3 (0.0–9.6)	904, 1.3 (0.0–9.3)	753, 1.2 (0.0–5.3)	2117, 1.4 (0.0–7.5)
Ferritin, ng/mL	232, 511.9 (25.3–2228.9)	97, 490.6 (26.8–2276.8)	41, 442.0 (27.0–1899.0)	37, 491.8 (31.6–1474.6)	57, 611.4 (20.4–2124.6)
Fibrinogen, mg/dL	353, 458.4 (181.8–812.0)	161, 472.4 (220.0–812.0)	80, 469.3 (182.8–800.3)	30, 450.4 (196.8–795.8)	82, 423.1 (184.1–735.0)
International normalized ratio	6977, 1.2 (0.9–3.0)	2586, 1.2 (0.9–3.0)	1258, 1.3 (1.0–3.4)	1058, 1.1 (0.9–2.2)	2075, 1.2 (0.9–2.9)
Lactate, mmol/L	6581, 2.1 (0.7–7.4)	2611, 2.2 (0.7–7.1)	1183, 2.1 (0.7–8.0)	773, 1.9 (0.7–6.1)	2014, 2.2 (0.7–7.9)
Lactate dehydrogenase, U/L	742, 377.4 (148.0–1169.8)	306, 361.2 (140.6–1025.8)	164, 352.7 (153.2–1048.3)	122, 493.5 (152.2–1873.0)	150, 342.8 (167.4–987.6)
Platelets, K/cu mm	26715, 244.6 (94.0–466.0)	9751, 245.7 (94.0–471.2)	4803, 242.9 (92.1–469.0)	3445, 235.9 (98.0–436.0)	8716, 247.7 (92.0–468.0)
Partial thromboplastin time, s	4030, 17.8 (0.8–43.0)	1473, 17.2 (0.8–43.8)	747, 16.9 (0.8–45.8)	646, 17.7 (0.8–41.8)	1164, 19.3 (0.8–40.2)
Troponin, N Tested, N Positive (%)	14,930, 1718 (4.6)	5395, 664 (4.6)	2699, 328 (4.6)	2056, 210 (4.9)	4780, 516 (4.5)
White blood cell count, K/cu mm	27,026, 15.7 (0.0–56.3)	9865, 14.9 (0.0–56.3)	4871, 21.0 (0.0–69.0)	3472, 15.2 (0.0–88.9)	8818, 14.0 (0.0–39.6)
Oxygen requirements, *N* (%)					
Low-flow oxygen, <2 L/min	2337 (6.3)	816 (5.7)	362 (5.1)	285 (6.6)	874 (7.7)
Mid-flow oxygen, 2–9 L/min	1769 (4.8)	660 (4.6)	333 (4.7)	266 (6.2)	510 (4.5)
High-flow oxygen, > 10 L/min	1278 (3.4)	453 (3.2)	210 (3.0)	138 (3.2)	477 (4.2)
Outcomes, N (%)					
Critical care outcome	3954 (10.7)	1523 (10.6)	742 (10.5)	406 (9.4)	1283 (11.3)
Inpatient care outcome	8343 (22.5)	3183 (22.2)	1577 (22.3)	1081 (25.0)	2502 (22.0)

Data are shown as frequencies with percentages in parentheses.

Models were prospectively evaluated in 15,670 ED visits by 14,103 unique patients, divided into two separate validation cohorts. The first included 4322 encounters that occurred during silent deployment of the CDS, and the second included 11,348 encounters that occurred after CDS was made viewable to ED clinicians (Fig. 1). Rates of critical care needs were 9.4% for the prospective silent and 11.3% for the prospective visible cohorts, while rates of inpatient care needs were 25.0% and 22.0%, respectively (Table 1).

All cohorts included representation of patients across age groups, gender, race, and ethnicity with most patients self-identifying as white non-Latino (38.0–43.7%) or black non-Latino (30.8–43.7%) and a minority of all cohorts identifying as Latino (8.5–15.8%). The five most common ED chief complaints across cohorts were shortness of breath, concern for COVID-19, chest pain, fever, and abdominal pain (Table 1). Prevalence of comorbidities, ED disposition vital signs, laboratory values, and oxygen requirements were similar across all time periods, as shown in Table 1. Rates of SARS-CoV-2 RT-PCR positivity were lower in our retrospective cohort (6.7%) than in our prospective cohort (17.4%) (Fig. 1). RT-PCR results were unknown at the time of ED disposition decision-making for a substantial portion of SARS-CoV-2 positive patients in all cohorts (Table 1).

### Model specification

Final models contained 39 distinct prediction variables that were normalized to discrete categories prior to input and inspected for relative importance to each model (Supplementary Table 1 and Supplementary Figure 1). Supplemental oxygen requirements and respiratory rates and trends were among the four most important predictors for both models. Shortness of breath was the only chief complaint that exhibited high importance for the prediction of both critical care and inpatient care outcomes. Levels of lactate, troponin, BUN, creatinine, AST, WBC and INR were important laboratory-based predictors. Histories of hypotension and kidney disease prior to the index visit were the only medical history elements among the 20 most important predictors for the critical care and inpatient care outcomes, respectively.

### Model performance

**Fig. 2:**
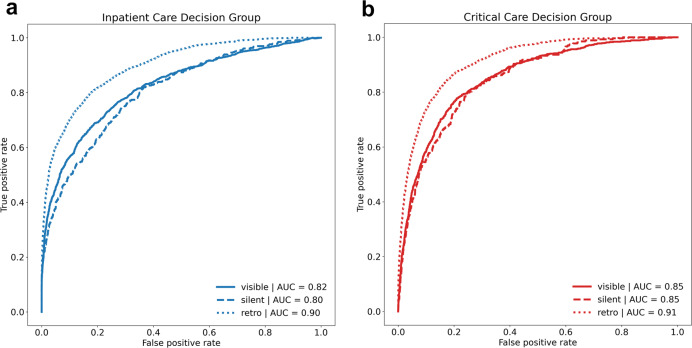
Model performance assessment. Receiver operating characteristic (ROC) curves are shown for our (**a**) inpatient care and (**b**) critical care outcome prediction models. ROC curves and measurements of area under the curve (AUC) are shown for three separate validation cohorts: retrospective out-of-sample (retro), prospective but prior to decision support activation (silent) and prospective after decision support activation (visible). Performance assessment was limited to patients not meeting outcome criteria prior to ED disposition decision.

Receiver operator characteristic curves for both models are shown in Fig. 2. Overall prediction performance within respective decision groups, as measured by area under the receiver operating characteristic curve (AUC) was robust under all scenarios. The critical care outcome prediction model achieved an AUC of 0.91 (95% CI 0.91–0.92) during derivation Fig. 2a) and 0.85 (95% CI 0.83–0.87) during the silent prospective validation. Predictive performance remained stable after model-driven CDS became visible to ED clinicians with an AUC of 0.85 (95% CI 0.84–0.87) (Fig. 2a). The inpatient care outcome prediction model achieved an AUC of 0.89 (95% CI 0.88–0.90) in our retrospective derivation cohort and 0.80 (95% CI 0.78–0.83) the prospective silent cohort. Predictive performance was similar after CDS was made visible in the clinical environment with an AUC of 0.82 (95% CI 0.81–0.84) (Fig. 2b). The Brier Score for this cohort was 0.080 and 0.129 for the critical care and acute care outcome, respectively. Calibration curves that by COVID-19 Deterioration Risk Level group may be seen in Supplementary Fig. 1.

### CDS Integration

**Fig. 3:**
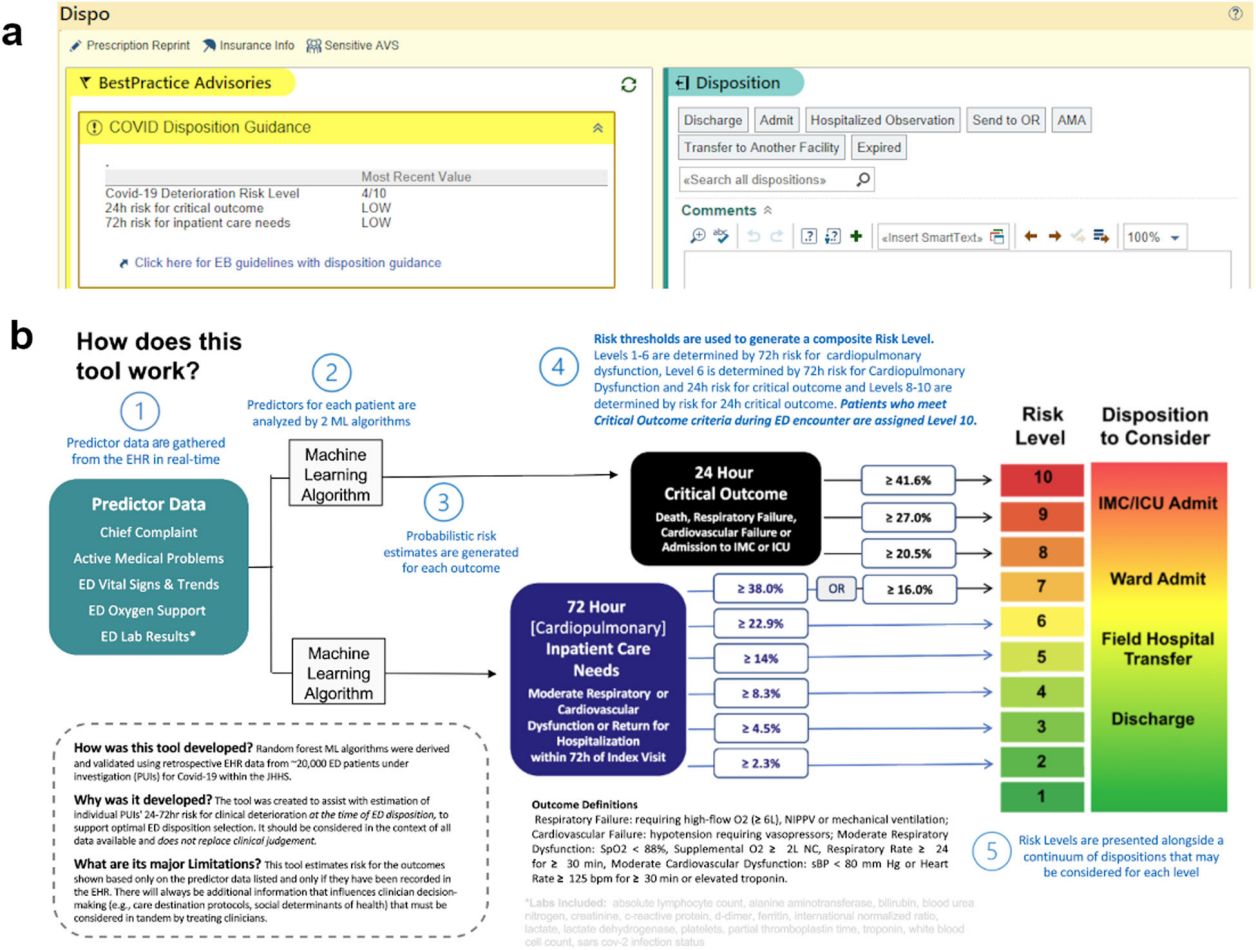
Clinical decision support interface. **a** Model-generated COVID-19 Deterioration Risk Levels were displayed in real-time for every patient with or under investigation for COVID-19 within the electronic health record (EHR). A screenshot of the emergency clinician disposition (Dispo) module is shown. **b** A hyperlink embedded within the Dispo module (bottom left of panel a) allowed emergency clinicians to access a more detailed explanation of model development and function within the EHR.

Model-generated outcome probabilities were translated to a single COVID-19 Clinical Deterioration Risk Level (1–10) for each ED encounter, which was then incorporated into EHR disposition workflow as a non-interruptive CDS module. COVID-19 Deterioration Risk Levels were displayed at the top of the ED clinician’s disposition module for all persons under investigation (PUIs) for COVID-19, along with estimated risk (low, moderate, high) of need for critical care within 24 h and need for inpatient care within 72 h (Fig. 3a). EHR-embedded hyperlinks were also provided, and allowed treating clinicians to access EHR-embedded web pages with additional model-driven disposition guidance and detailed information about model derivation, validation and risk-thresholding schema (based on observed risk) used to generate risk levels (Fig. 3b). As shown in Fig. 3b, assignment to levels 1–6 was determined based on model-estimated risk of inpatient care outcome at 72 h, while assignment to levels 8–10 was assigned based on model-estimated risk of critical care outcome at 24 h. Assignment to level 7 could be assigned based on meeting the risk threshold for either (or both) outcome.

### Patient-oriented outcomes

**Fig. 4:**
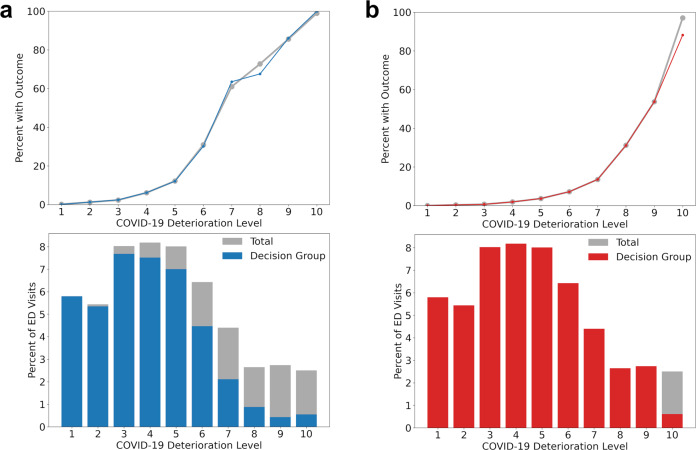
Distribution of ED visits across risk levels (bottom panel) and percent of patients within each risk level who met outcome criteria (top panel) during the index hospital visit are shown for the (**a**) inpatient care and (**b**) critical care outcome models. Data for the decision group only are shown in solid colors (blue and red) and data for all patients are shown in gray.

The distribution of ED visits across COVID-19 Deterioration Risk Levels during the prospective visible portion of our study, along with the proportion of visits where patients met inpatient care and/or critical care outcome criteria, is shown in Fig. 4. Overall rates of hospitalization, mortality, 24-h ICU upgrade, 72-h ED return, and lengths of stay were similar during the retrospective and prospective visible study periods (Table 2). A reduction in mortality from 6.7% (95% CI 5.7–7.8%) to 2.9% (95% CI 1.9–3.8%) was observed among high-risk patients (level 9–10) following CDS deployment. Nonsignificant downward trends in rate of 24-h ICU upgrade were also seen for high risk (11.4 [95% CI 10.0–12.8%] before versus 8.5% [95% CI 6.9–10.2%] after) and elevated risk patients (level 7–8) patients (5.9% [95% CI 5.0–6.9%] versus 3.9% [95% CI 2.7–5.1%]).

**Table 2:** Patient-oriented outcome measures.

	All patients	High risk (9–10)	Elevated risk (7–8)	Moderate risk (4–6)	Low risk (1–3)
Total, No					
Retrospective	22,347	2163	2909	9328	7947
Prospective visible	11,348	1214	1200	4995	3939
Hospitalized, No, % (95% CI)					
Retrospective	9661, 43.2 (42.6–43.9)	2001, 92.5 (91.4–93.6)	2428, 83.5 (82.1–84.8)	4515, 48.4 (47.4–49.4)	717, 9.0 (8.4–9.7)
Prospective visible	4757, 41.9 (41.0–42.8)	1065, 87.7 (85.9–89.6)	953, 79.4 (77.1–81.7)	2242, 44.9 (43.5–46.3)	497, 12.6 (11.6–13.7)
24-h Mortality, No, % (95% CI)					
Retrospective	158, 0.7 (0.6–0.8)	146, 6.7 (5.7–7.8)	7, 0.2 (0.1–0.4)	5, 0.1 (0.0–0.1)	0, 0.0 (0.0–0.0)
Prospective visible	47, 0.4 (0.3–0.5)	35, 2.9 (1.9–3.8)	6, 0.5 (0.1–0.9)	5, 0.1 (0.0–0.2)	1, 0.0 (0.0–0.1)
24-h ICU Upgrade, No, % (95% CI)					
Retrospective	475, 4.9 (4.5–5.3)	229, 11.4 (10.0–12.8)	144, 5.9 (5.0–6.9)	92, 2.0 (1.6–2.4)	10, 1.4 (0.5–2.3)
Prospective Visible	206, 4.3 (3.8–4.9)	91, 8.5 (6.9–10.2)	37, 3.9 (2.7–5.1)	61, 2.7 (2.0–3.4)	17, 3.4 (1.8–5.0)
72-h ED Return, No, % (95% CI)					
Retrospective	623, 5.6 (5.1–6.0)	4, 4.7 (0.2–9.1)	28, 8.0 (5.2–10.9)	298, 7.0 (6.3–7.8)	293, 4.5 (4.0–5.0)
Prospective visible	296, 5.9 (5.2–6.5)	5, 18.5 (3.9–33.2)	14, 9.7 (4.9–14.6)	150, 7.0 (5.9–8.0)	127, 4.7 (3.9–5.5)
Total LOS hospitalized, Median (IQR)					
Retrospective	98 (49–178)	146 (73–268)	121 (70–213)	81 (39–147)	29 (8–91)
Prospective visible	97 (47–172)	141 (77–264)	110 (62–188)	84 (30–147)	52 (9–106)
Total LOS discharged, Median (IQR)					
Retrospective	4 (2–6)	5 (3–62)	5 (3–11)	4 (3–6)	3 (1–5)
Prospective visible	5 (3–7)	5 (3–7)	5 (3–8)	5 (4–7)	4 (3–6)

*ED* emergency department, *ICU* intensive care unit, *LOS* length of stay.

## Discussion

In this study, we developed and deployed an EHR-embedded CDS system that relied on locally derived ML prediction models to optimally match PUIs for COVID-19 with appropriate care and guidance. Our models reliably estimated risk for short-term clinical deterioration and model output was translated into actionable advice within existing clinical workflow for emergency clinicians.

While numerous other ML prediction and prognostication models for COVID-19 have been reported^9,10^, very few have been integrated into clinical care or used to guide health system resource utilization. Indeed, clinical implementation of artificial intelligence assisted tools (including ML) has been slow in general, despite a proliferation of studies describing their development in many fields of medicine. Translation of ML models to clinical practice is difficult for reasons both technical (e.g., suboptimal prospective performance, difficult to integrate into existing EHR workflow) and social (e.g., fear of replacement, lack of trust)^11,12^. These challenges are accentuated in the ED practice environment, where decisions must be made rapidly and with limited information.

We overcame these barriers by employing a pragmatic and user-centered approach to data science and CDS development. Unlike previously reported ML models for COVID-19, ours were designed to be used not only for patients with known COVID-19 infection, but also in those where it is suspected. Infection status is often unknown at the point of ED disposition decision-making (Table 1) and models developed for prognostication of patients with confirmed infection only are of limited utility to ED clinicians. Infection status (positive, negative, unknown) was included as a predictor in our models but carried much less importance than direct measurements of a patient’s condition (e.g., vital signs, shortness of breath) and trends. Our outcomes were designed by clinicians to capture physiologic states and events that reflect specific care level (eg, inpatient, ICU) needs, as these are the events ED clinicians are attempting to anticipate when making disposition decisions.

Our approach to model training and evaluation was also unique. Many patients met full or partial outcome criteria at the time of ED disposition; the traditional approach is often to exclude these patients from all cohorts. However, these patients represent valid cases for algorithmic learning because they are part of the spectrum of illness severity (e.g., highest severity) seen in the ED. The ML methods applied can draw relationships between outcome and predictor data useful for predictive modeling. These patients were included in training datasets but were excluded from reported predictive performance metrics in testing and validation datasets. These exclusions were made to isolate the patients where disposition decisions may be usefully supported by CDS (i.e., decision group). This modeling and validation approach is purposeful in evaluating the pragmatic utility of CDS in practice.

Our models also provide insights that can inform clinical practice and interpretation of similar models in the future. Diabetes, chronic lung disease, cardiovascular disease and hypertension have all been identified as important risk factors for severe illness and mortality due to COVID-19^13,14^, yet these comorbidities were not among the most important predictors for either of our models (Supplementary Fig. 1). Age and gender, two demographic variables that have consistently been linked to risk for adverse COVID-19 outcomes^14,15^, also carried relatively little weight in our models. While these findings may seem counterintuitive, they likely reflect the reality that in acute care environments, physiologic manifestations of disease may be even more important indicators of near-term clinical trajectory than epidemiologic risk factors^16,17^. Indeed, most predictors with high importance across both our models were either direct (e.g., respiratory rate and blood pressure) or indirect (*eg*, shortness of breath and creatinine level) measures of pathophysiologic state at the time of prediction. Our findings are aligned with those of others, including Wongvibulsin *et al* who reported that markers of respiratory status comprised the five most important predictors in an ML model designed to estimate 1-day risk of severe COVID-19 or death in inpatients^18^. Similarly, Haimovich *et al* found supplemental oxygen needs and SpO_2_ were the most important predictors in an ML model designed to identify ED patients at risk of progression to critical COVID-19 respiratory illness within 24 h and reported that reliable risk estimates for this outcome could be made using respiratory rate, supplemental oxygen flow rate and SpO_2_ alone^19^.

This study does have limitations. First, ML models were derived and validated using data from a single health system. This weakness was minimized via use of large training and testing datasets, the inclusion of ED encounters from a variety of practice settings (community and academic, urban, and suburban) and the use of two prospectively collected datasets for secondary validation. Translation of our work to other settings would require re-training and re-validation of models using locally derived datasets. This limitation must be considered for all ML-assisted technology applied to data from the EHR, as algorithms developed and validated in one clinical context are unlikely to be immediately transportable to another^20^. In addition, our CDS system was deployed system-wide to optimize clinical care and healthcare resource utilization under pandemic conditions. To mitigate untoward effects that could have been caused by degradation of model performance in the face of an evolving virus and rapidly changing approaches to combating COVID-19 (e.g., antivirals, steroids, vaccination), we performed extensive post-implementation surveillance including frequent checks of model performance and found them to be stable over time and across vaccinated and unvaccinated populations. We believe this was due to the large-scale data available for model building, the ML approaches deployed to minimize over-fitting, and our direct measurement of physiologic state (predictor variables) that are known to relate to demands for care regardless of underlying mechanisms. Finally, the lack of a controlled study design and confounding pandemic-related changes in ED and hospital operations also limited our ability to systematically assess the impact of our intervention. We did monitor clinician behaviors and several patient-oriented outcomes closely throughout the study and observed a general trend toward improvement but cannot conclude reliably that this was due to our CDS system alone.

## Methods

### Setting and selection of participants

This study was performed at five EDs within a university-based health system between 3-1-2020 and 7-20-2021. Study sites included two urban academic EDs (Johns Hopkins Hospital (JHH) and Bayview Medical Center (BMC)) and three suburban community EDs (Howard County General Hospital (HCGH), Suburban Hospital (SH), and Sibley Memorial Hospital (SMH)) with a combined patient volume of 270,000 visits per year. All adult patients (≥18 years old) designated as PUIs for COVID-19 were included in the study; COVID-19 infection status was not used as an inclusion criterium because ED disposition decisions are often made before infection status is known and because others who have tested negative continue to be treated as PUIs based on elevated clinical suspicion and presumption of a false negative result^21^. PUI status was operationally defined as having active isolation orders in the EHR at the time of ED disposition. Patients who were not under suspicion for COVID-19, including those who underwent asymptomatic testing for SARS-CoV-2, were excluded.

Our retrospective model building cohort (i.e., derivation cohort) was comprised of ED visits that occurred between 3-1-2020 and 11-15-2020 at all five sites. Models were prospectively validated using data collected between 11-25-2020 and 7-20-2021, with performance measured and reported separately for periods when model-driven CDS was silent (not visible) and available for use by treating ED clinicians.

### Methods of measurement

Outcome and predictor data were extracted from the EHR (Epic, Verona, WI). Candidate predictor variables were identified by comprehensive review of preprint and peer-reviewed literature on COVID-19 and were evaluated by clinicians and data scientists for face validity and collection reliability; variables were incorporated into final models based on univariate assessment of their relationship to outcomes (e.g., descriptive statistics and graphical plots) and their additive value to ML model predictive performance (differences in AUC)^22–28^. The objective was to achieve high predictive performance with a parsimonious ML model that also considered the constraints and reliability of real-time data feeds. To ensure model output was optimized to the decision we aimed to support, ML prediction time-points were set as the time of first disposition order entry (e.g., discharge or hospitalization orders) for each patient.

### Outcome and predictor measures

The primary outcomes predicted were critical care needs and inpatient care needs within 24 and 72 h of ED disposition, respectively. Outcome definitions were developed by consensus among a committee of attending physicians in emergency medicine (JH and GK), internal medicine (TD and AS), and critical care medicine (DH and RSS). Criteria for critical care were met if a patient died, was admitted to an intermediate or intensive care unit, or developed cardiovascular or respiratory failure within 24 h of ED disposition. Cardiovascular failure was defined by hypotension requiring intravenous vasopressor support (dopamine, epinephrine, norepinephrine, phenylephrine or vasopressin). Respiratory failure was defined by hypoxia or hypercarbia requiring high-flow oxygen (>10 liters/minute), high-flow nasal canula, noninvasive positive pressure ventilation or invasive mechanical ventilation^19^. Criteria for inpatient care needs were met if patients exhibited at least moderate cardiovascular dysfunction (systolic blood pressure <80 mmHg, heart rate ≥125 for ≥30 min or any troponin measurement >99^th^ percentile), respiratory dysfunction (respiratory rate ≥24, hypoxia with documented SpO2 < 88% or administration of supplemental oxygen at a rate >2 liters/minute sustained for ≥30 min) or were discharged at initial ED visit and had a return ED visit and hospitalization within 72 h. Prediction horizons (24 h for critical care needs and 72 h for inpatient care needs) were selected to guide decision-making related to disposition and level of care determinations. Patients discharged without meeting outcome criteria before reaching 24 or 72 h were assumed to be outcome negative.

Data used for prediction were limited to those routinely stored in the EHR during ED care. To be included in analysis, predictor data had to be recorded and available in the EHR prior to the time of prediction. Data elements included patient demographics (age, sex), chief complaint(s), active medical problems (identified based on ICD-10 codes), vital signs, routine laboratory results, markers of inflammation (c-reactive protein [CRP], d-dimer, ferritin), SARS-CoV-2 status, and respiratory support requirements.

Predictor data were prepared as categorical variables. Continuous variables (e.g., lab results, vital signs) were transformed to discrete categories to enable representation of predictor missingness. The pre-model fit processing for each type of data was performed as follows. Demographics (age, gender) were input as categories with age grouped in 10-year increments^29^. Chief complaint(s) were limited to a structured pick-list (819 complaints) and grouped into clinically meaningful categories as described previously^30–33^. Active medical problems (ICD-10 codes) were grouped as binary features (present vs. not present) for atrial fibrillation, coronary artery disease, cancer, cerebrovascular disease, diabetes, heart failure, hypertension, immunocompromised, kidney disease, liver disease, pregnancy, prior respiratory failure, and smoking. Vital signs were discretized as normal or gradations of abnormal based on physiology-based criteria^34,35^. The latest vital signs recorded prior to ED disposition were included as predictors along with a comparison to the initial triage vitals (prior to ED interventions) to characterize vital trends (e.g., stable, trending normal, trending abnormal). Laboratory data were characterized as not resulted (0), resulted within the normal range (1) and resulted with relevant gradations of abnormal (e.g., 2–4). The SARS-CoV-2 status predictor was classified as unknown or SARS-CoV-2 positive. Respiratory support was categorized as no oxygen, low-flow (≤2 L/min), mid-flow (>2 and <10 L/min) or high-flow (see above) prior to disposition decision^19^. The exact form of each predictor variable, including discretization of continuous variables and the treating of missingness, is detailed in Supplementary Table 1.

### Model derivation

The retrospective derivation cohort was randomly divided into training (two-thirds) and testing (one-third) datasets. Separate ensemble-based decision tree learning algorithms (random forest^36^) were trained to predict each outcome (critical care needs within 24 h, inpatient care needs within 72 h). During training, the random forest algorithm (number of estimators = 50, minimum leaf size = 10) executed a randomized sampling process to train a set of individual decision trees and aggregated output to produce a single probabilistic prediction for each outcome^37^. To maximize opportunity for algorithmic learning, all encounters by PUIs were included in training datasets, including those where patients met criteria for the outcome of interest prior to the point of prediction. Performance of each model was evaluated in test sets using the subset of patients for whom model-driven decision support was relevant at the point of decision-making. This subset was termed the ‘decision group’ and was defined separately for each outcome. For the critical care outcome, the decision group included all patients who had not met any outcome criteria (cardiopulmonary failure or death) prior to the time of ED disposition decision (identified by time of order entry). For the inpatient care outcome within 72 h, the decision group included patients who did not meet any outcome criteria at the time of ED disposition decision; patients who met pre-specified criteria for cardiopulmonary dysfunction early in their ED visit but whose dysfunction had resolved by the time of ED disposition decision were included in this group. Patients not belonging to decision groups were excluded from testing datasets. Multiple model performance measures were applied during model derivation and prospective evaluation. Receiver operating characteric (ROC) curve analysis was performed, which included measuring the Area Under the ROC Curve (AUC) with 95% confidence interval estimates calculated using Delong’s method^38,39^. Meausurements of COVID-19 Deterioration Risk Level distribution and associated outcome probability were reported. Overall goodness-of-fit (Brier Score) and calibration curves (plots of observed versus predicted risk) were evaluated^40^. Model interpretation was perfomed using feature importance measures including SHapley Additive exPlanations (SHAP) values to assess predictor impact and directionality^41^.

### Model validation

Models underwent prospective validation using a similar approach. Prospective model performance was measured and reported separately for a cohort of ED visits that occurred while our CDS system operated silently and had no impact on clinical care delivery and for visits that occurred after CDS was made visible to ED clinicians. As described for the testing dataset of our retrospective cohort, prospective performance was measured and reported for patients belonging to the decision only (those not already meeting outcome criteria at the time of disposition).

### Clinical decision support system development

A system to generate patient-level risk estimates and deliver EHR-embedded CDS to emergency clinicians in real-time was developed with software engineers and end-users under a human-centered design framework. ML models were triggered to generate new outcome risk estimates each time new predictor data (e.g., vital signs, laboratory results) were filed to the EHR. To facilitate rapid interpretation at the point-of-care, model-generated outcome probabilities were translated to one of ten COVID-19 Deterioration Risk Levels using risk thresholding; thresholds were determined by consensus between technical and clinical team members using graphical plots, calibration curves, and outcome frequency tables. Thresholds were designed based on the objective to distribute COVID-19 Deterioration Risk Levels over a 1 (low risk) to 10 (high risk) scale using the observed probability of each outcome. Brief non-interruptive CDS that contained risk levels was populated within existing EHR workflow (i.e., disposition module) for eligible patients only, with more elaborate CDS made available via an EHR-embedded hyperlink. CDS content and appearance was developed iteratively, guided by direct feedback from prospective end-users.

### Clinical implementation and ongoing quality assurance

Our CDS system was activated to operate silently, suppressed from ED clinician view, beginning on 11-25-2020. COVID-19 Deterioration Risk Levels and associated CDS became viewable in each participating ED serially between 12-8-2020 and 2-23-2021 (JHH 12-8-2020; BMC 12-22-2020; HCGH 1-13-2021; SH 2-17-2021; SMH 2-23-2021) and remained viewable until 7-20-2021. Prospective model performance, patient distribution across risk levels and patient-oriented outcomes including rates of hospital admission, ICU admission (direct and secondary due to escalation of care within 24 h), 24-h mortality and 72-h ED return for discharged patients were monitored regularly during the pre- and post-implementation periods. CDS performance and patient outcome reports were made available to clinical and hospital IT leadership teams at each site. All measures were reported separately for silent and visible periods.

Before CDS was made viewable, all ED clinicians at each site received live training on the purpose and function of the system by an ED clinician study team member (JH or AM). They were also provided recorded materials for asynchronous study and review via email and EHR-embedded hyperlinks. Training sessions included detailed explanations of ML model function (outcomes, predictors and algorithmic processing), model performance, and emphasis of the continued importance of clinician judgement in individual patient assessment.

Data infrastructure, ML prediction models and CDS software were developed and evaluated under the approval of the Johns Hopkins Medicine Institutional Review Board (IRB00185078).

### Reporting summary

Further information on research design is available in the Nature Research Reporting Summary linked to this article.

## Supplementary information

Supplemental Information

Reporting Summary

## Data Availability

The clinical data used in this study are from the Johns Hopkins Health System (JHHS). These individual-level patient data are protected for privacy. Qualified researchers affiliated with Johns Hopkins University (JHU) may apply for access through the Johns Hopkins Institutional Review Board (IRB) (https://www.hopkinsmedicine.org/institutional_review_board/). Those not affiliated with JHU seeking to collaborate may contact the corresponding author. Access to these data for research collaboration with JHU must ultimately comply with IRB and data sharing protocols (https://ictrweb.johnshopkins.edu/ictr/dmig/Best_Practice/c8058e22-0a7e-4888-aecc-16e06aabc052.pdf).

## Code availability

All analyses were performed using Python 3.6. The code used to develop and evaluate prediction models and generate risk-levels is available in a public repository (https://github.com/CDEM-JHU/COVID19-ADMIT-CDS). This code relies on open-source libraries, most notably scikit-learn (https://scikit-learn.org/stable/) and shap (https://shap.readthedocs.io/en/latest/index.html). The code that supports data collection, cleaning, normalization and quality control made use of proprietary data structures and libraries, so we are not releasing this code. However, details of the precise implementation are available in the methods section, supplementary material, and public repository to allow for independent replication.
