# Supplementary material for: Multisite implementation of a workflow-integrated machine learning system to optimize COVID-19 hospital admission decisions
Source: NPJ Digit Med. 2022 Jul 16;5:94. doi: 10.1038/s41746-022-00646-1 (PMC9287691; doi:10.1038/s41746-022-00646-1)
Supplement: Supplementary file 1 — Supplemental Information [file 41746_2022_646_MOESM1_ESM.pdf]

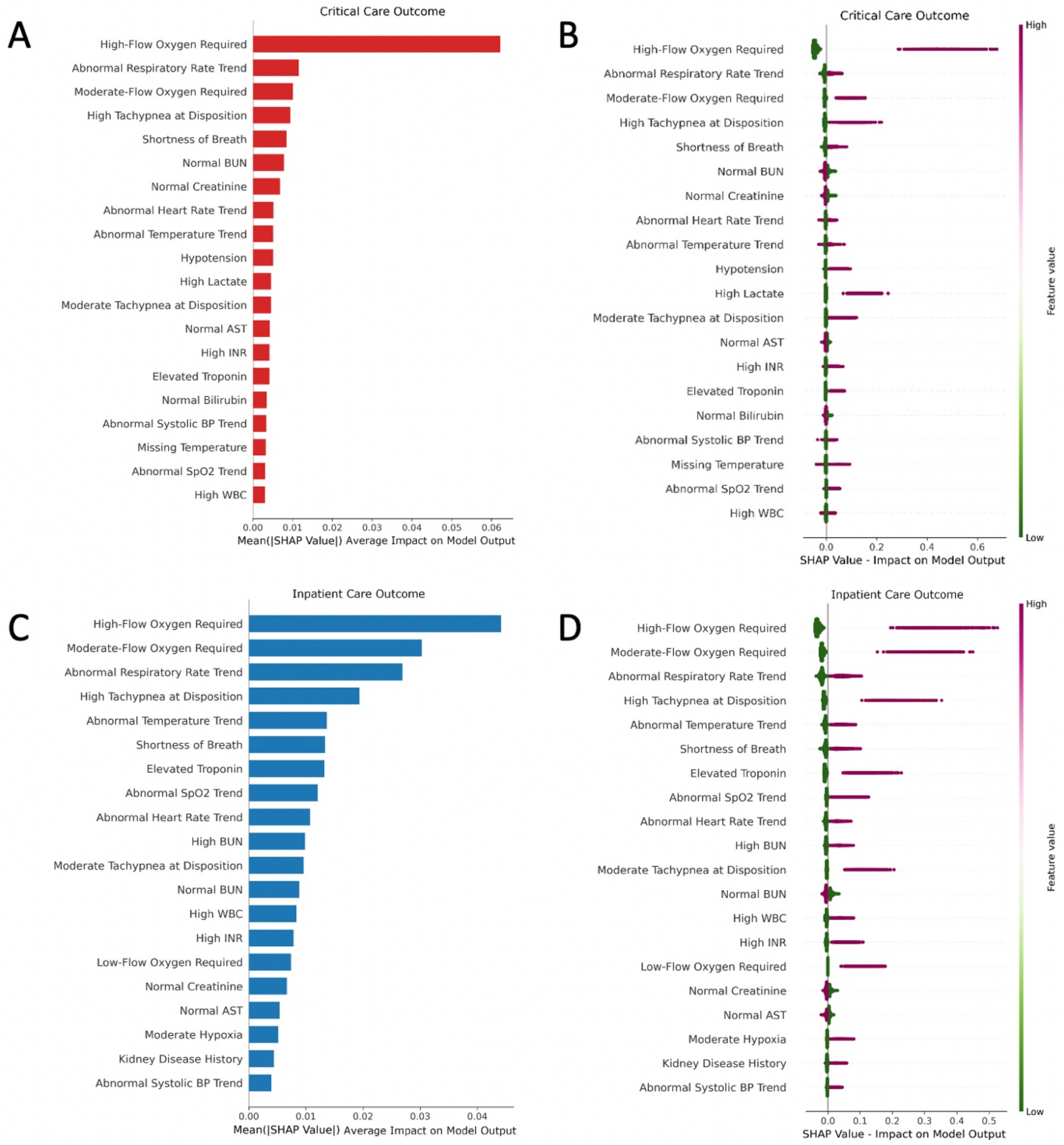


**Supplementary Figure 1.** Shapley Additive exPlanations (SHAP) for the 20 most impactful predictors in the model for the critical care (A and B) and acute care (C and D) outcomes in the prospective (visible) cohort. A and C shows the average predictor impact on each model output ranked from most to least impact (mean absolute SHAP values). B and D shows Beeswarm plots of SHAP values where each point corresponds to an individual patient. The color represents the categorical variable value; a variable that occurred (i.e., binary 1) is purple and a variable that did not occur (binary = 0) is green. A negative SHAP value (extending left) indicates a decreased probability of outcome and a positive SHAP value (extending) right indicates an increased probability of outcome. For example high tachypnea at disposition was impactful in increasing the probability of both the critical care (Panel B) and inpatient care (Panel D) outcomes. AST: Aspartate Aminotransferase; BUN: blood urea nitrogen; INR: international normalized ratio; WBC: white blood cell count

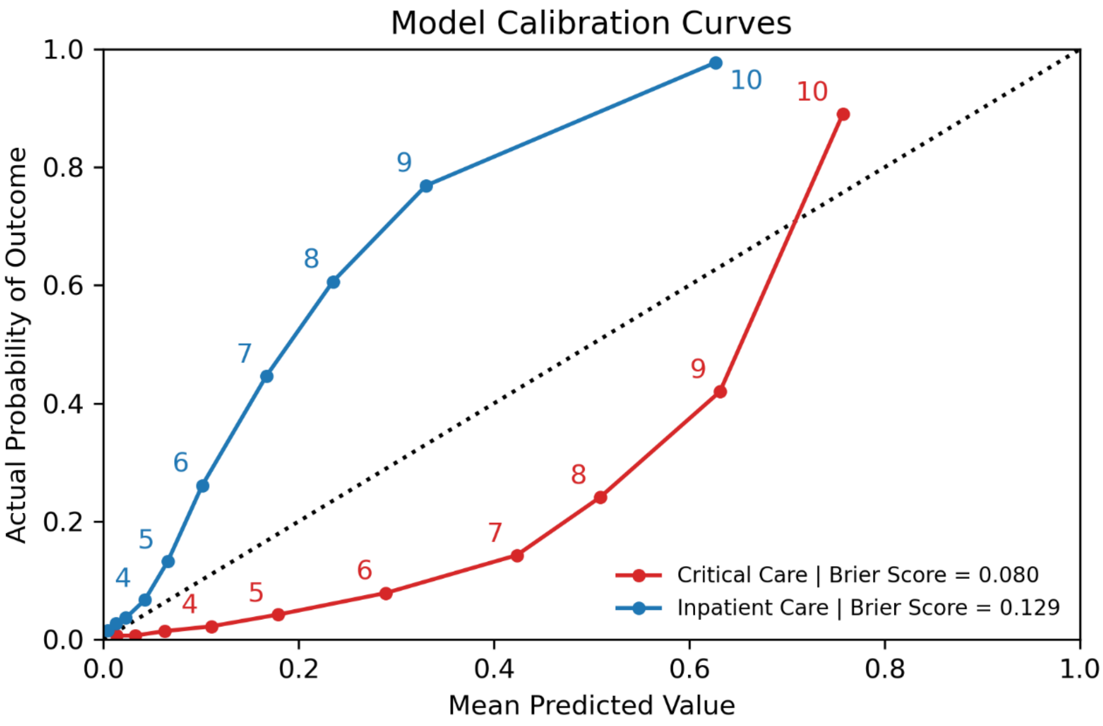


**Supplementary Figure 2.** Calibration curves along with Brier Scores for the critical care (red) and acute care (blue) models. Point labels indicate the COVID-19 Deterioration Risk Level group

## Supplementary Table 1

| Predictors | Predictor Categories | Predictor Form |
|---|---|---|
| **Demographics** | | |
| Age | 18-29; 30-39; 40-49; 50-59; 60-69; 70-79; 80-89; >=90 | Discretized. |
| Gender | Female, Male | |
| **Complaints** | | |
| Complaint Groups | Abdominal pain; Abnormal Findings; Abscess; Allergic; Altered mental status; Arrest; Autoimmune; Back pain; Blunt trauma; Burn; Cancer; Cardiovascular; Chest pain; Collision; Constitutional symptoms; COVID19; Dental; Device; Dialysis; Dysrhythmia; Edema; Endocrine; Ear nose throat; Environmental; Epistaxis; Fever; General; Genitourinary; GI bleeding; Glucose abnormal; Head trauma; Headache; Hematological; Hypertension; Hypotension; Lower respiratory tract infection; Mass; Medication management; Musculoskeletal isolated trauma; Musculoskeletal non-trauma; Neurological; Nausea vomitting diahrea; Opthamologic; Penetrating trauma; Pregnancy; Psychological; Referral; Seizure; Shortness of breadth; Sickle cell; Skin nails and hair; Social issues; Substance abuse; Syncope; Transplant; Upper respiratory tract infection; Weakness; Wound; Wound Check; Surgical Wound | Captured as absent or present. Groups are non-mutually exclusive. Patients may have multiple complaints. |
| **Vital Signs** | | |
| Temperature, °F | Hypothermic (<95); Modertate Hypothermia (95-96.2); Normal (96.3-99.2); Moderate Hyperthermia (99.3-100.5); Hypothermic (≥ 100.6); Missing | Discretized latest vital sign prior to ED disposition. Discretization includes a missing category. |
| Heart Rate, bpm | Severe Bradycardia (<50); Mild Bradycardia (50-59); Normal (60-104); Mild Tachycardia (105-109); Moderate Tachycardia (110-119); High Tachycardia (120-129); Severe Tachycardia (> 130); Missing | |
| Respiratory Rate, bpm | Hyponea (<14); Mild Hypopnea (14-15); Normal (16-19); Mild Tachypnea (20-22); Moderate Tachypnea (23-27); High Tachypnea (28-29); Severe Tachypnea (≥30); Missing | |
| Oxygen Saturation, % | Severe Hypoxia (<90); Moderate Hypoxia (90-94); Normal (95-100); Missing | |
| Systolic Blood Pressure, mmHg | Hypotension (<100); Mild Hypotension (100-107); Normal (108-176); Mild Hypertension (177-199); Hypertension (> 200); Missing | |
| **Vital Signs Trends** | | |
| Temperature | Stable normal; Trending abnormal; Trending normal; Abnormal stable | Discretized trend from triage vital to disposition vital quantified in patterns of: Stable normal* = normal to normal; Trending abnormal = normal-to-abnormal; Trending normal = abnormal-to-normal; Abnormal stable = abnormal-to-abnormal. |
| Heart Rate | Stable normal; Trending abnormal; Trending normal; Abnormal stable | |
| Respiratory Rate | Stable normal; Trending abnormal; Trending normal; Abnormal stable | |
| Oxygen Saturation | Stable normal; Trending abnormal; Trending normal; Abnormal stable | |
| Systolic Blood Pressure | Stable normal; Trending abnormal; Trending normal; Abnormal stable | |
| **Labs** | | |
| Absolute Lymphocyte Count, K/cu mm | Low (<0.8); Normal (≥0.8); Not Resulted | Discretized latest laboratory result prior to ED disposition. Discretization includes a not resulted (missing) category. |
| Alanine Aminotransferase, U/L | Normal (<40); High (≥ 40); Not Resulted | |
| Aspartate Aminotransferase, U/L | Normal (<40); High (≥ 40); Not Resulted | |
| Biliriubin, mg/dl | Normal (<1.3); High (≥ 1.3); Not Resulted | |
| Blood Urea Nitrogen, mg/dL | Normal (<22); High (≥ 22); Not Resulted | |
| Creatinine, mg/dL | Normal (<1.3); High (≥ 1.3); Not Resulted | |
| C-Reactive Protein, mg/dL | Normal (<10); High (> 10); Not Resulted | |
| D-Dimer, mg/L | Normal (<1); Elevated (1-1.9); High (≥ 2); Not Resulted | |
| Ferritin, ng/mL | Normal (<300); High (≥ 300); Not Resulted | |
| Fibrinogen, mg/dL | Low (<150); High (≥150); Not Resulted | |
| International Normalized Ratio | Normal (<1.1); High (≥ 1.1); Not Resulted | |
| Lactate, mmol/L | Normal (<2); Elevated (2-3.9); High (≥ 4); Not Resulted | |
| Lactate Dehydrogenase, U/L | Normal (<250); High (≥ 250); Not Resulted | |
| Platelets, K/cu mm | Low (<150); Normal (≥150); Not Resulted | |
| Partial Thromboplastin Time, sec | Normal (<1.3); High (≥ 1.3); Not Resulted | |
| Troponin, ng/mL | Normal (<0.04); High (≥ 0.04); Not Resulted | |
| White Blood Cell Count, K/cu mm | Normal (<4); Elevated (4-11.9); High (≥ 12); Not Resulted | |
| SARS-CoV-2 | Unknown (Under Investigation); Positive | |
| **Oxygen** | | |
| Oxygen Requirements, L/minute | No oxygen; Low-Flow (<2); Mid-Flow (2-9); High-Flow (≥ 10) | Discretized as highest level of oxygen support required prior to ED disposition. |
| **Active Problems** | | |
| Co-Morbidity Groups | Atrial fibrilation; Coronary artery disease; Cancer; Cerebrovascular disease; Diabetes; Heart failure; Hypertension; Immunosuppression; Kidney disease; Lung disease (e.g., COPD); Pegnancy, Prior respiratory failure; Smoking | Captured as absent or present. Groups are non-mutually exclusive. |

** Definitions of normal and abnormal vitals are listed in undere Discrete Categories within the Vital Signs at ED Disposition section.*
