# Supplementary material for: Multisite implementation of a workflow-integrated machine learning system to optimize COVID-19 hospital admission decisions
Source: NPJ Digit Med. 2022 Jul 16;5:94. doi: 10.1038/s41746-022-00646-1 (PMC9287691; doi:10.1038/s41746-022-00646-1)
Supplement: Supplementary file 2 — Reporting Summary [file 41746_2022_646_MOESM2_ESM.pdf]

nature portfolio

Corresponding author(s): Jeremiah Hinson, MD, PhD
jhinson4@jhmi.edu

Last updated by author(s): Apr 28, 2022

Reporting Summary

Nature Portfolio wishes to improve the reproducibility of the work that we publish. This form provides structure for consistency and transparency in reporting. For further information on Nature Portfolio policies, see our Editorial Policies and the Editorial Policy Checklist.

Statistics

For all statistical analyses, confirm that the following items are present in the figure legend, table legend, main text, or Methods section.

n/a	Confirmed	
☐	☒	The exact sample size (n) for each experimental group/condition, given as a discrete number and unit of measurement
☐	☒	A statement on whether measurements were taken from distinct samples or whether the same sample was measured repeatedly
☐	☒	The statistical test(s) used AND whether they are one- or two-sided<br>*Only common tests should be described solely by name; describe more complex techniques in the Methods section.*
☐	☒	A description of all covariates tested
☐	☒	A description of any assumptions or corrections, such as tests of normality and adjustment for multiple comparisons
☐	☒	A full description of the statistical parameters including central tendency (e.g. means) or other basic estimates (e.g. regression coefficient) AND variation (e.g. standard deviation) or associated estimates of uncertainty (e.g. confidence intervals)
☐	☒	For null hypothesis testing, the test statistic (e.g. F, t, r) with confidence intervals, effect sizes, degrees of freedom and P value noted<br>*Give P values as exact values whenever suitable.*
☒	☐	For Bayesian analysis, information on the choice of priors and Markov chain Monte Carlo settings
☐	☒	For hierarchical and complex designs, identification of the appropriate level for tests and full reporting of outcomes
☒	☐	Estimates of effect sizes (e.g. Cohen's d, Pearson's r), indicating how they were calculated

Our web collection on statistics for biologists contains articles on many of the points above.

Software and code

Policy information about availability of computer code

Data collection	All data were collected from a relational database (Clarity) that underlies the electronic medical record (Epic) of our health system. Code used for data queries is available from the corresponding author upon reasonable request.
Data analysis	All analyses were performed using Python 3.6. The Python code for the analyses is available from the corresponding author upon reasonable request.

For manuscripts utilizing custom algorithms or software that are central to the research but not yet described in published literature, software must be made available to editors and reviewers. We strongly encourage code deposition in a community repository (e.g. GitHub). See the Nature Portfolio guidelines for submitting code & software for further information.

Data

Policy information about availability of data

All manuscripts must include a data availability statement. This statement should provide the following information, where applicable:

- Accession codes, unique identifiers, or web links for publicly available datasets
- A description of any restrictions on data availability
- For clinical datasets or third party data, please ensure that the statement adheres to our policy

The clinical data used in this study are from patients within the Johns Hopkins Health System (JHHS). These individual-level data are protected for privacy. Qualified researchers that are part of Johns Hopkins University may apply for access through the Institutional Review Board.

Field-specific reporting

Please select the one below that is the best fit for your research. If you are not sure, read the appropriate sections before making your selection.

☒ Life sciences ☐ Behavioural & social sciences ☐ Ecological, evolutionary & environmental sciences

For a reference copy of the document with all sections, see nature.com/documents/nr-reporting-summary-flat.pdf

Life sciences study design

All studies must disclose on these points even when the disclosure is negative.

Sample size

All encounters that met study inclusion criteria during our study periods were included to maximize sample size, power and opportunity for algorithmic learning.

Data exclusions

To maximize opportunity for algorithmic learning, all encounters by patients under investigation for COVID-19 (PUIs) were included in training datasets, including those where patients met criteria for the outcome of interest prior to the point of prediction. PUI status was operationally defined as having active isolation orders in the EHR at the time of ED disposition. Patients who were not under suspicion for COVID-19, including those who underwent asymptomatic testing for SARS-CoV-2, were excluded.

Performance of each model was evaluated in test sets using the subset of patients for whom model-driven decision support was relevant at the point of decision-making. This subset was termed the ‘decision group’ and was defined separately for each outcome. For the critical care outcome, the decision group included all patients who had not met any outcome criteria (cardiopulmonary failure or death) prior to the time of ED disposition decision (identified by time of order entry). For the inpatient care outcome within 72 hours, the decision group included patients who did not meet any outcome criteria at the time of ED disposition decision; patients who met pre-specified criteria for cardiopulmonary dysfunction early in their ED visit but whose dysfunction had resolved by the time of ED disposition decision were included in this group. Patients not belonging to decision groups were excluded from testing datasets.

Replication

Model performance was validated prospectively in two separate data sets (pre- and post-deployment of CDS). Performance in each validation dataset is reported.

Randomization

There was no randomization.

Blinding

Blinding was not relevant, as there were not two arms. Models were derived and validated using all patient encounters and CDS was deployed to support clinical care of all patients. Outcomes were defined using EHR surveillance criteria as above and researchers had no ability to alter this at the patient level.

Reporting for specific materials, systems and methods

We require information from authors about some types of materials, experimental systems and methods used in many studies. Here, indicate whether each material, system or method listed is relevant to your study. If you are not sure if a list item applies to your research, read the appropriate section before selecting a response.

Materials & experimental systems

n/a	Involved in the study	
☒	☐	Antibodies
☒	☐	Eukaryotic cell lines
☒	☐	Palaeontology and archaeology
☒	☐	Animals and other organisms
☐	☒	Human research participants
☐	☒	Clinical data
☒	☐	Dual use research of concern

Methods

n/a	Involved in the study	
☒	☐	ChIP-seq
☒	☐	Flow cytometry
☒	☐	MRI-based neuroimaging

Human research participants

Policy information about studies involving human research participants

Population characteristics

This was an EHR-based study that included clinical data from ED encounters by PUIs for COVID-19 (see above for further details). After models were derived and validated, decision support was deployed to support care under pandemic conditions as part of a system-wide quality improvement initiative. We have included characteristics of the patient population in Table 1 of our manuscript.

Recruitment

There was no recruitment

Ethics oversight	Data infrastructure, ML prediction models and CDS software were developed and evaluated under the approval of the Johns Hopkins Medicine Institutional Review Board (IRB00185078).

Note that full information on the approval of the study protocol must also be provided in the manuscript.

Clinical data

Policy information about clinical studies

All manuscripts should comply with the ICMJE guidelines for publication of clinical research and a completed CONSORT checklist must be included with all submissions.

Clinical trial registration	This was not a clinical trial.
Study protocol	This was not a clinical trial, but all details of model derivation and validation, decision support development and deployment and patient-oriented outcome monitoring are provided in our manuscript.
Data collection	We analyzed data from 37,212 adult encounters at five emergency departments within a university-based health system between 3-1-2020 and 7-20-2021.
Outcomes	The primary outcomes predicted were critical care needs and inpatient care needs within 24 and 72 hours of ED disposition, respectively. Outcome definitions were developed by consensus among a committee of attending physicians in emergency medicine, internal medicine, and critical care medicine. Criteria for critical care were met if a patient died, was admitted to an intermediate or intensive care unit, or developed cardiovascular or respiratory failure within 24 hours of ED disposition. Cardiovascular failure was defined by hypotension requiring intravenous vasopressor support (dopamine, epinephrine, norepinephrine, phenylephrine or vasopressin). Respiratory failure was defined by hypoxia or hypercarbia requiring high-flow oxygen (>10 liters/minute), high-flow nasal canula, noninvasive positive pressure ventilation or invasive mechanical ventilation. Criteria for inpatient care needs were met if patients exhibited at least moderate cardiovascular dysfunction (systolic blood pressure < 80 mmHg, heart rate >125 for >30 minutes or any troponin measurement >99th percentile), respiratory dysfunction (respiratory rate >24, hypoxia with documented SpO2 <88% or administration of supplemental oxygen at a rate >2 liters/minute sustained for >30 minutes) or were discharged at initial ED visit and had a return ED visit and hospitalization within 72 hours. Prediction horizons (24 hours for critical care needs and 72 hours for inpatient care needs) were selected to guide decision-making related to disposition and level of care determinations. Patients discharged without meeting outcome criteria before reaching 24 or 72 hours were assumed to be outcome negative.<br><br>Data used to compute these outcomes were collected from a relational database (Clarity) that underlies the electronic medical record (Epic) of our health system.
